# The immunogenicity and protection efficacy evaluation of mRNA vaccine candidate for severe fever with thrombocytopenia syndrome in mice

**DOI:** 10.1371/journal.pntd.0012999

**Published:** 2025-04-30

**Authors:** Da-Eun Jeong, Jack Yoon, Baek Kim, Jun-Gu Kang

**Affiliations:** 1 Korea Zoonosis Research Institute, Jeonbuk National University, Iksan, Republic of Korea; 2 Vernagen LLC, Tucker, Georgia, United States of America; Faculty of Science, Ain Shams University (ASU), EGYPT

## Abstract

Severe fever with thrombocytopenia syndrome virus (SFTSV) is a tick-borne viral pathogen that causes Severe fever with thrombocytopenia syndrome (SFTS) in humans with a high fatality rate. Currently, there are no approved antivirals or vaccines against SFTSV. The envelope protein of SFTSV, which consists of two domains, Gn and Gc, has been investigated as a target antigen for the development of SFTSV vaccines. Here, we used an mRNA platform to develop an effective and safe SFTSV vaccine. Our mRNA vaccine candidate harbored an equal number of mRNAs individually encoding the full-length SFTSV Gn or Gc domain. These mRNAs were produced using a 5′ cap, SmartCap, by *in vitro* transcription and then packaged with an ionizable lipid nanoparticle platform, called STLNP. Robust expression of these Gn and Gc antigens was observed when human 293 T cells were transfected with the SFTSV mRNA formulation. When mice were immunized with our SFTSV vaccine candidate, the collected serum displayed strong immunogenicity and *in vitro* neutralization activity against SFTSV. Thus, the immunized mice showed complete protection with a lethal dose of SFTSV, without any pathological traces of SFTSV infection-mediated tissue damage. Thus, our mRNA vaccine platform is a promising SFTS vaccine candidate for clinical development.

## Author summary

Severe fever with thrombocytopenia syndrome (SFTS) is a zoonosis disease with a high fatality rate in East Asia including Republic of Korea (ROK). Although the fatality rate of SFTS has increased since its first discovery, there are no available vaccines or treatments for SFTS. In the present study, we developed mRNA vaccine candidates encoding the glycoproteins (Gn and Gc) of the SFTS virus (SFTSV), which are major targets for neutralizing antibodies. We evaluated the efficacies of SFTS mRNA vaccine candidates (2 μg, 10 μg, and 20 μg of VER-001) using C57BL/6 and IFNAR^-/-^ mice. VER-001 showed induction of high immunogenicity and complete protective efficacy without body weight loss in the immunized mice. Therefore, our study showed that all doses of VER-001 effectively induce antibody formation and inhibit lethal SFTSV infection and are potential vaccine candidates.

## Introduction

The first case of severe fever with thrombocytopenia syndrome (SFTS) was reported in China in 2010 [1]. Since then, patients with SFTS have been reported in Japan, the Republic of Korea (ROK), and Taiwan [2–4]. SFTS is a zoonotic infectious disease caused by the SFTS virus (SFTSV), which belongs to the *Bandavirus* genus of the *Phenuiviridae* family. The clinical symptoms of SFTS include high fever, thrombocytopenia, leukocytopenia, and gastrointestinal and neurological symptoms [5]. SFTSV is a negative-sense single-stranded RNA virus consisting of three segments: large (L), medium (M), and small (S). These segments encode the RNA-dependent RNA polymerase (RdRp), viral envelope glycoproteins (glycoprotein N-terminus, Gn; glycoprotein C-terminus, Gc), nucleocapsid protein (NP), and non-structural protein (NS) [6]. Among these components, Gn and Gc, which correspond to the M segment, N-terminal, and C-terminal of the SFTSV glycoprotein, are produced through the proteolytic cleavage of the glycoprotein precursor. Also, glycoproteins represent heterodimers formed on the surface of the virus, which are crucial for cell attachment and membrane fusion, facilitating host cell entry and are considered major targets for neutralizing antibodies [7–11].

SFTSV is a tick-borne virus that can be transmitted from animals to humans, although its general transmission occurs through tick vectors [12,13]. The fatality rate of SFTS is notably high, accounting to 18.7% in the Republic of Korea, 5.3% in China, and 23% in Japan [14–16]. However, no effective antiviral therapies or vaccines are currently available for treating SFTSV infections. Therefore, vaccines and treatments are necessary. Various vaccine candidates for SFTS, including DNA vaccines [17,18], recombinant virus vaccine [19,20], subunit vaccine [21,22] and viral vectors [23], inactivated virus [24], and mRNA vaccine [25] have been explored.

The mRNA-lipid nanoparticle (LNP) technology has been widely used in various viral vaccine studies since the development of the first mRNA vaccine candidate in 1993. The approval process for mRNA vaccine platforms has been hindered due to the stability of mRNAs caused by extracellular RNases and various age-related issues. However, the development of stable mRNA delivery systems, such as lipid nanoparticles (LNP), has been a significant breakthrough. In response to the COVID pandemic in 2021, Pfizer-BioNTech and Moderna created their own mRNA vaccines for SARS-CoV-2, which were approved [26]. Among the approved mRNA-LNP vaccines, ionizable LNP are used as delivery systems to induce a strong immune response. LNP encapsulate and protect mRNAs and effectively deliver them to dendritic and target cells for antigens [27–29]. The mRNA-LNP vaccine platform has been used to develop vaccines targeting a variety of viruses, including severe acute respiratory syndrome coronavirus-2 (SARS-CoV-2) [30–33], Zika virus [34], Influenza virus [35], Chikungunya virus [36], Monkeypox virus [37], and more. The mRNA vaccine platform produces target antigen protein(s) in host cells, which can induce an antigen-specific and strong immune response similar to actual viral infections [38]. Moreover, mRNA vaccines have numerous advantages such as scalability, rapid production, and the ability to stimulate robust neutralizing antibodies and T-cell responses, as demonstrated by the recent COVID mRNA vaccines [39,40]. For example, the approved Moderna mRNA vaccine showed high immune response induction and complete protection efficacy and demonstrated safety with respect to mRNA vaccines [41]. The mRNA vaccine platform has been validated for its advantages such as low manufacturing costs, high capacity for antibody formation, and stability [42,43].

Experimental animal models are essential for understanding SFTSV pathogenicity and developing antiviral treatments or vaccines [44]. Despite SFTSV’s ability to infect various species, including dogs, cats, pigs, horses, poultry, and goats, most immunocompetent experimental animal models do not exhibit severe disease symptoms [45–49]. Therefore, researchers have developed SFTSV-susceptible models using type 1 interferon receptor knockout (IFNAR^-/-^) mice, STAT2-deficient golden Syrian hamsters, and aged ferrets. IFNAR^-/-^ mice are immunodeficient and cannot induce a complete immune response against viral infections. Therefore, the IFNAR^-/-^ mouse serves as one of the valuable infection animal models for evaluating the protective efficacy of vaccines against lethal viruses [50,51]. Additionally, the IFNAR^-/-^ mouse is commonly used for drug testing and various other research purposes [52,53].

In this study, we evaluated the immunogenicity and protective efficacy of an SFTSV mRNA vaccine candidate (VER-001), which contains two full-length Gn and Gc protein-encoding mRNAs encapsulated in LNPs, in the C57BL/6 and IFNAR^-/-^ mouse models. We observed that VER-001 effectively protected immunized mice against a lethal dose challenge with SFTSV without any clinical symptoms, pathological impacts, body weight loss, or death. Thus, VER-001 is a promising vaccine candidate against SFTSV infections.

## Materials and methods

### Ethics statement

Animal studies were performed in an Animal Biosafety Level 3 facility at the Korea Zoonosis Research Institute. This study was approved by the Korea Zoonosis Research Institute and the Institutional Animal Care and Use Committee of Jeonbuk National University (JBNU 2022-062). All animal experiments were performed strictly according to national guidelines and animal suffering was minimized.

### Virus preparation

Vero - E6 cells (ATCC-CRL1586) were grown in Dulbecco’s modified Eagle’s medium (DMEM, Welgene, Kyeongsan, ROK) which included 10% fetal bovine serum (FBS; Welgene) and 1% penicillin/streptomycin (Welgene) at 37°C and 5% CO_2_. The SFTSV 2015-JJ01 strain (NCBI Accession numbers: MN329148-MN329150) was cultured in Vero-E6 cells for 5 days. The supernatant of the infected cell was harvested 5 days post-infection and stored at -80°C. Virus titration was confirmed using a focus reduction neutralization test (FRNT_50_). Monolayer of Vero E6 cells were infected using stored virus stock and incubated at 37°C for 2 h. After removing the supernatant containing the infected virus, the cells were washed once with Dulbecco’s phosphate-buffered saline (DPBS, Welgene) and then incubated in overlay media ((DMEM containing 10% FBS and 2% methyl cellulose (Sigma-Aldrich, St. Louis, MO, USA)). After six days, the cells were fixed with 4% paraformaldehyde (CellNest, Hanam, Korea) and washed three times with DPBS. The cells were incubated with rabbit anti-SFTSV NP antibody (Abclon, Seoul, ROK) at a dilution of 1:1,000, followed by incubation with horseradish peroxidase (HRP)-conjugated goat anti-rabbit IgG antibody (Enzo, Farmingdale, NY, USA). The cells were stained using 3,3′-diaminobenzidine tetrahydrochloride (Sigma-Aldrich).

### Preparation of mRNA template (Plasmid DNA and Linearized DNA) for manufacturing

Plasmid DNA was prepared using an EndoFree Plasmid Maxi Kit (Cat.12362, QIAGEN, Hilden, Germany) according to the manufacturer’s protocol. Approximately, 100 µg of supercoiled DNA, SFTSV-Gc and SFTSV-Gn were linearized using BspQI restriction enzyme (NEB, Ipswich, MA, USA) at 50°C for 2 h.

### Formulation of mRNA-LNP (STL1244)

STL1244 cells were prepared as previously described [54]. Briefly, LNPs were prepared by mixing lipid components and an aqueous mRNA solution using a microfluidic device (Nanoassembly Ignite + , Precision Nanosystems, Canada). A novel ionizable lipid, STP1244 (chemically synthesized by ST Pharm), DOPE (helper lipid, Merck, Darmstadt, Germany), cholesterol (structural lipid, Sigma-Aldrich), and C16 PEG2000-Ceramide (PEG-lipid, Avanti Polar Lipids, Alabaster, AL, USA) were mixed at a specific molar ratio of lipid molecules: DOPE: cholesterol: C16 PEG2000-Ceramide = 36.5: 15: 52:1.5 (Mol%). The lipid components dissolved in ethanol and mRNA in 100 mM sodium acetate buffer were mixed in a volume ratio of 1:1 and formulated at a flow rate of 12 mL/min. The obtained LNPs were diafiltrated using 1 × PBS and concentrated using tris buffer with sucrose by ultrafiltration (Amicon Ultra-15 Centrifugal Filter Unit, Sigma-Aldrich). The concentrated LNPs were stored at -20°C until further use. To measure mRNA concentration and encapsulation efficiency (%), a Ribogreen assay (Thermo Fisher Scientific, Waltham, MA, USA) was performed using a spectrofluorometer (FP-8350, Jasco, Tokyo, Japan). The size distribution and zeta potential of the well-dispersed LNPs in 1 × PBS were measured using a dynamic light scattering (DLS) instrument (Zetasizer Ultra, Malvern, UK) with a 173° scattering angle at room temperature.

### In vitro transcription for manufacturing

In vitro transcription was performed using a mixture of 5 mM of ATPsol, CTPsol, GTPsol, and m1UTPsol (N1-methyl pseudo UTP solution), 1 × T7 RNA polymerase buffer, 4 mM SmartCap SC101, 20 µg of linearized plasmid DNA, 0.8 U of YPP, 320 U of RRI, and 16.3 KU of T7 RNA polymerase to obtain a total volume of 400 μl with NFW at 42°C ± 1°C for 4 h. Plasmid template was removed by adding 160 U DNase I at 37°C ± 1°C for 1 h. The synthesized mRNA was purified from residual reagents by centrifugation at 6,000 g, 16°C for 10 min using the Amicon Ultra tube (Cat. UFC210096, 30 K, Merck Millipore, Burlington, MA, USA). After repeated washing until the A260/230 value was 2.0 or higher, the filter was inserted upside down into the new tube and the centrifugation step was repeated at 6,000 g, 16°C for 2 min. To check the quality, the mRNA was diluted to 200 ng and stored in a refrigerator at 75°C for 4 min and 4°C for 5 min before loading onto 1% agarose gel.

### *In Vitro* Transcription (IVT)

DNA template sequence for mRNA *in vitro* transcription (IVT) consisted of T7 promoter, 5′ untranslated region (UTR), open reading frame of Gn or Gc modified from the SFTSV membrane glycoprotein DNA (GenBank: KP663735.1; KAGWH3 strain), 3′ UTR and 120 bases of poly adenine (polyA). The 5′ UTR and 3′ UTR are from the beta globin gene of *Xenopus laevis*. The DNA fragment was synthesized and subcloned into a pUC57-Kan vector using GenScript (Piscataway, NJ, USA).

The plasmid vector was linearized using the restriction enzyme BspQI (New England Biolabs, Ipswich, MA, USA) to obtain SFTSV Gn and Gc. N1-Methylpseudouridine (m1Ψ) was purchased from Advent Bio (IL, USA). IVT condition was performed according to the manufacture’s recommendation (TranscriptAid T7 High Yield Transcription Kit, ThermoFisher, Waltham, MA, USA): ATP/CTP/GTP/m1ψTP: (5 mM each), SmartCap (SC101, 4mM, ST Pharm, Seoul, ROK), Linear template DNA (1 μg of plasmid or 0.5 μg of PCR product), and T7 RNA polymerase enzyme mix (2 μl) was used for the experiment. IVT was carried out in a 20 μl reaction mixture incubated at 37°C for 2 h. The template DNA was removed using 2 units of DNase I (ThermoFisher) and treated at 37°C for 15 min post which, column purification (Monarch RNA Cleanup Kit, New England Biolabs).

### Transfection

One microgram of mRNAs was transfected into 293FT (Thermo Fisher Scientific) or SJCRH30 (CRL-2061, ATCC, MD, USA) cells in a 12 well plate using Lipofectamine MesseangerMax (Thermo Fisher Scientific) at a ratio of 1:2 according to the manufacturer’s protocol.

### Western blot

Cell pellets were lysed with NP-40 lysis buffer (150 mM sodium chloride/1% NP-40/50 mM Tris, pH 8.0) after 24 h of transfection. The cell lysates were harvested after centrifugation and loaded onto the SDS-PAGE gel (Bio-Rad, CA, USA) and transferred onto a 0.45 μm PVDF membrane (Bio-Rad). The PVDF membranes were blocked with 1% bovine serum albumin (Sigma-Aldrich, MO, USA) in tris-buffered saline (TBS, Biosesang, Yongin, ROK) buffer containing 0.1% Tween 20 (TBST, Daejung, Siheung, ROK) for 1 h at room temperature. The membranes were washed 3 times with 1 × TBST and incubated for 2 h with rabbit anti-Gn antibody (NBP2-41153, Novus Biologiclas) and anti-Gc antibody (NBP2-41156, Novus Biologiclas, WA, USA, 1:1,000 in TBST) and anti-GAPDH (sc-47724, Santa Cruz, TX, USA, 1:1,000 in TBST) overnight at 4°C. The membranes were then washed three times with 1 × TBST and incubated with horseradish peroxidase-conjugated secondary antibody (HAF007 and HAF008, R&D Systems, 1:1,000 in TBST) for 1 h at room temperature. The membranes were washed three times with 1 × TBST and SuperSignal West Pico Plus Chemiluminescent Substrate (Thermo Fisher Scientific) was used for protein detection.

### Mouse immunization and SFTSV challenge

Female C57BL/6 wild-type mice aged 8 weeks were intramuscularly immunized with empty LNP and three doses (2 μg, 10 μg, and 20 μg) of VER-001 were administered twice at 3-week intervals. The sera of immunized mice were collected four times: one week, one month, two months, and three months after the last inoculation. Approximately, 8-week-old female IFNAR^-/-^ mice were immunized with empty LNP and two doses of VER-001 and then challenged with 1 × 10^3^ PFU of SFTSV 2 weeks after the final immunization. Body weights and survival rates were monitored in an Animal Biosafety Level 3 facility at the Korea Zoonosis Research Institute. The mice were sacrificed 2 and 4 days post-infection (dpi), and their organs (liver, spleen, and kidneys) and whole blood were collected for viral quantification using qRT-PCR.

### Enzyme-Linked Immunosorbent Assays (ELISA)

ELISA was used to measure SFTSV IgG titers specific to SFTSV Gn (Immune Technology, Tarrytown, NY, USA) and Gc (Immune Technology). The 96-well plates (Sarstedt, Numbrecht, Germany) were coated overnight at 4°C with 100 ng/well purified Gn and Gc protein (1 mg/ml) in coating buffer [Na_2_CO_3_H_2_O (Kanto chemical, Tokyo, Japan) and H_2_CO_3_ (Junsei, Gimhae, ROK), pH 9.6]. After 16 h, the plates were washed twice with PBST (0.05% Tween 20 (Daejung) in 1 × PBST). After washing, the plates were blocked at room temperature for 2 h with 5% skim milk (BD Difco, Sparks, MD, USA) in PBST and washed twice with PBST. The plates were serially diluted with mouse serum samples (1:100), incubated at room temperature for 1.5 h and washed 4 times with PBST. Subsequently, the serum samples were analyzed using horseradish peroxidase-conjugated goat anti-mouse IgG (1:10,000; Enzo). Plates were then washed 4 times with PBST, 3,3′,5,5′-tetramethylbenzidine peroxidase substrate solution (Biolegend, San Diego, CA, USA) was added and it was incubated for 20 min. The reaction was stopped by adding 1M H_3_PO_4_ solution (Daejung) and the plates were read using a microplate reader at 450 nm.

### Focus reduction neutralization test (FRNT_50_)

The neutralization activity of sera from immunized mice was determined using the focus reduction neutralization test (FRNT_50_). Approximately, 1 × 10^5^ Vero E6 cells were seeded into 24 well plates. (Sarstedt, Numbrecht, Germany) After overnight incubation at 4°C, serum in serial dilution of 1:40, 1:160, 1:640, and 1:2560 were mixed with equal volumes of diluted 1,000 PFU virus. The mixtures were incubated at 4°C for 1 h with mixing. The mixture was added to 24 well plate and incubated at 37°C and the plate was gently inverted for 2 h. Mixed media was then removed and 1 ml of overlay media was added at 37°C for 6 days. After six days, the plates were fixed with 4% paraformaldehyde (CellNest) for 1h. The fixed cells were washed three times with PBS. Cells were incubated with rabbit anti-SFTS NP antibody (1:1,000 dilution in 1% BSA; MPbio, Santa Ana, CA, USA) and followed by goat anti-HRP rabbit IgG antibody (1:1,000 dilution in 5% skimmilk). The cells were stained using 3,3′-diaminobenzidine tetrahydrochloride (Sigma-Aldrich).

### Flow cytometric analysis

Flow cytometric analysis was conducted to quantitate IFN-γ secreting CD4 and CD8 positive T cell response. Spleens were collected from immunized mice and grinded through the 70 μm cell strainer (BD biosciences). After grinding splenocytes and red blood cell lysis with Red Blood Cell Lysing Buffer Hybri-Max (Sigma), splenocytes were cultured for 16 h in RPMI medium containing 10% FBS and 1% penicillin/streptomycin and stimulated with 10 μg of purified Gn and Gc antigens in 96 well V-bottomed culture plates (Nunc, Roskilde, Denmark). To detect the expression of intracellular IFN-γ, cells were treated with 1 μg Golgiplug (BD Bioscience), ionomycin, and PMA 4 h prior to staining. Cells were then blocked with superblock solution (10% rat sera, 10% hamster sera, and 10% mouse sera, Sigma) for 30 min at 4°C and stained with surface marker, anti-CD3 (145-2c11) (BD Bioscience), CD4 (RM4-59, BD Bioscience), and CD8 (53-6.7, Biolegend). After single cell staining, the cells were fixed and permeabilized using the Cytofix/perm kit (BD Bioscience) for 20 min at 4°C. Fixed cells were washed twice and stained with intracellular cytokines for 30 min at 4°C, then, analyzed using a CytoFLEX S flow cytometer (Berkman Coulter Inc, Brea, CA, USA). The detected cells were analyzed using the FlowJo software version 10.6 (Treestar, Ashland, OR, USA).

### Quantitative reverse transcription-polymerase chain reaction (qRT-PCR)

Total RNA was extracted from the tissues and whole blood of infected mice using TRIzol (Favorgen, Ping-Tung, Taiwan) and TRIzol LS reagent (Invitrogen, MA, USA). Tissues mixed with TRIzol were homogenized using TissueLyser II (Qiagen, Hilden, Germany) and were extracted. Viral RNA was quantified using a DiaStar OneStep Multiplex qRT-PCR kit (Solgent, Daejeon, Republic of Korea). The qRT-PCR was performed using the Quant studio 3 (Thermo fisher Scientific, MA, USA) under the following settings: reverse transcription at 50°C for 30 min, enzyme activation at 95°C for 15 min, denaturation at 95°C for 20 sec, annealing and extension at 60°C for 40 sec and 40 cycles of amplification. The qRT-PCR was derived from the NP gene of SFTSV: F-(5′-CCTTCAGGTCATGACAGCTGG-3′) and R-(5′-ACCAGGCTCTCAATCACTCCTGT-3′) and probe (5′-6FAM-AGCACATGTCCAAGTGGGAAGGCTCTG-BHQ1–3′). Viral copy numbers were calculated and compared with those of the standard controls.

### Hematoxylin and eosin staining (H&E staining)

Hematoxylin and eosin (H&E) staining was performed to confirm necrosis, white pulp formation, and infiltration of infected mouse organ cells. Livers, kidney, and spleens of sacrificed mice were fixed in 10% formalin for 24 h at 4°C. Tissues of organs were cut to obtain 4 μm-thick sections and stained with hematoxylin and eosin. The stained tissues were analyzed for infiltration of inflammatory, necrotic cells, and white pulp in each organ. using an Olympus CX43 microscope. Each section was scored by a trained staff member.

### Statistical analysis

All data were statistically analyzed using Prism8 and Excel. The *p*-values were calculated using two-way ANOVA and a two-tailed Mann-Whitney U test. Results are represented as mean ± S. D. The *p*-values of *p *≤ 0.05 were considered significant.

## Results

### Generation of SFTS mRNA vaccine candidate (VER-001)

**Fig 1:**
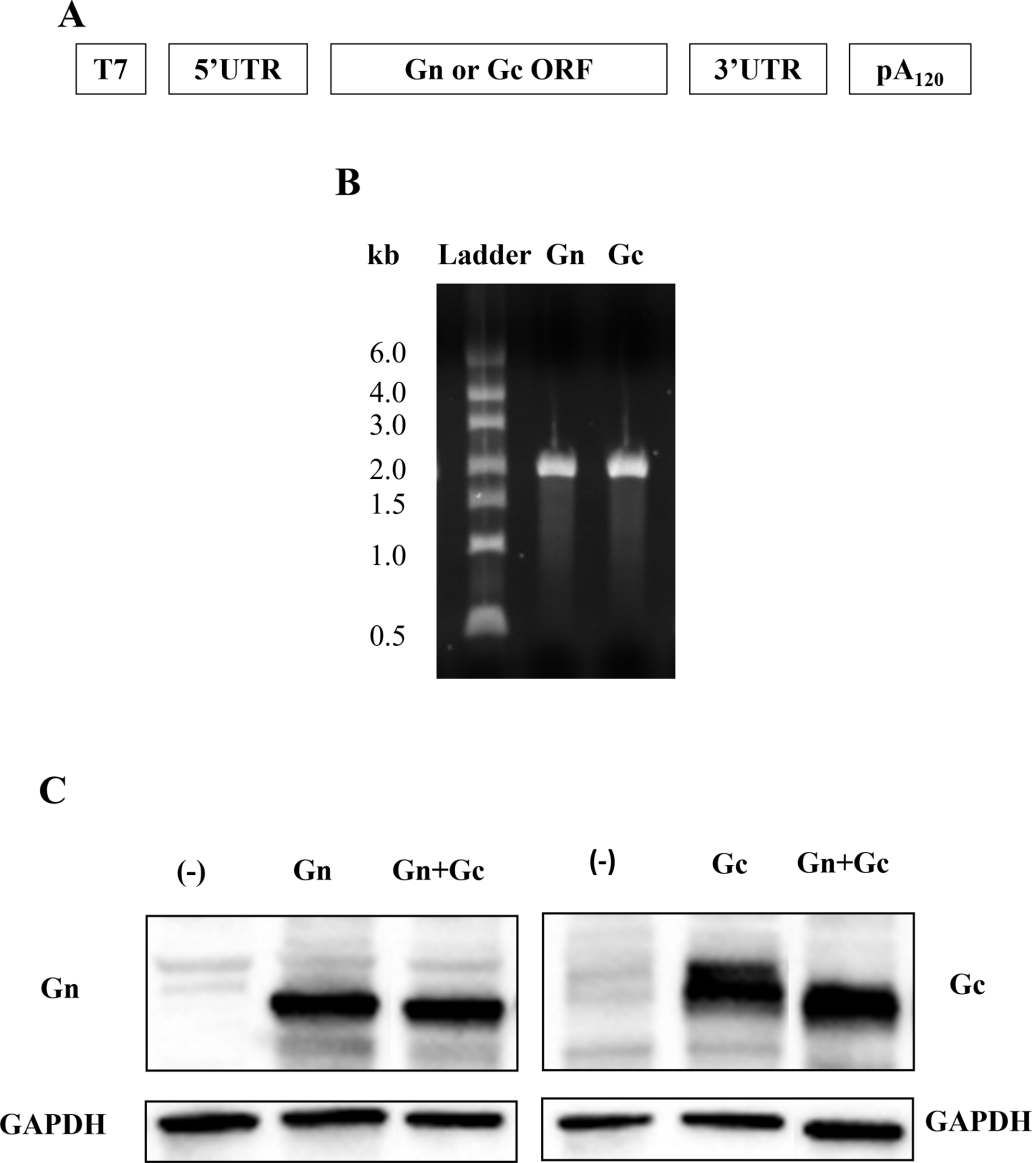
Generating constructs of mRNA-Gn-Gc vaccine candidates. **(A)** Gn and Gc mRNA structure from DNA template with T7 promoter includes 5′ and 3′ UTRs and 120 nt of polyadenine. **(B)** RNAs from in vitro transcription. In vitro transcribed Gn and Gc mRNA were analyzed using 1% agarose gel (E-Gel EX Agarose Gels, Invitrogen). **(C)** Western blot analysis of Gn and Gc mRNA expression in 293FT cells. Approximately, 1 µg of individual Gn or Gc mRNA, or both were transfected into 293FT cells and their expression was analyzed through immunoblot after 24 h of transfection.

VER-001, a newly developed SFTS mRNA vaccine candidate, was designed to include two mRNAs that individually express the Gn and Gc proteins, which are widely recognized as the major antigenic components of the SFTSV envelope glycoprotein and facilitate host cell attachment and entry [55]. The two mRNAs of VER-001 were produced from the plasmids that encode T7 promoter, 5′ UTR, the SFTSV antigen genes (Gn or Gc), 3′ UTR, and poly-adenylation (> 110 rAs) by T7 RNA polymerase-mediated *in vitro* transcription (Fig 1A and 1B). To detect the expression of Gn and Gc antigen proteins in the produced mRNAs, 293FT cells were transfected with Gn or Gc mRNA, or both. As shown in Fig 1C, we confirmed the successful expression of Gn and Gc proteins by western blot analysis using cell lysates of the transfected cells collected 24 h post-transfection (Fig 1C). Finally, Gn and Gc mRNAs were encapsulated in an ionizable LNP (STLNP) for VER-001 formulations designed for vaccine efficacy tests in mice.

### VER-001 elicited strong humoral immune response in mice

**Fig 2:**
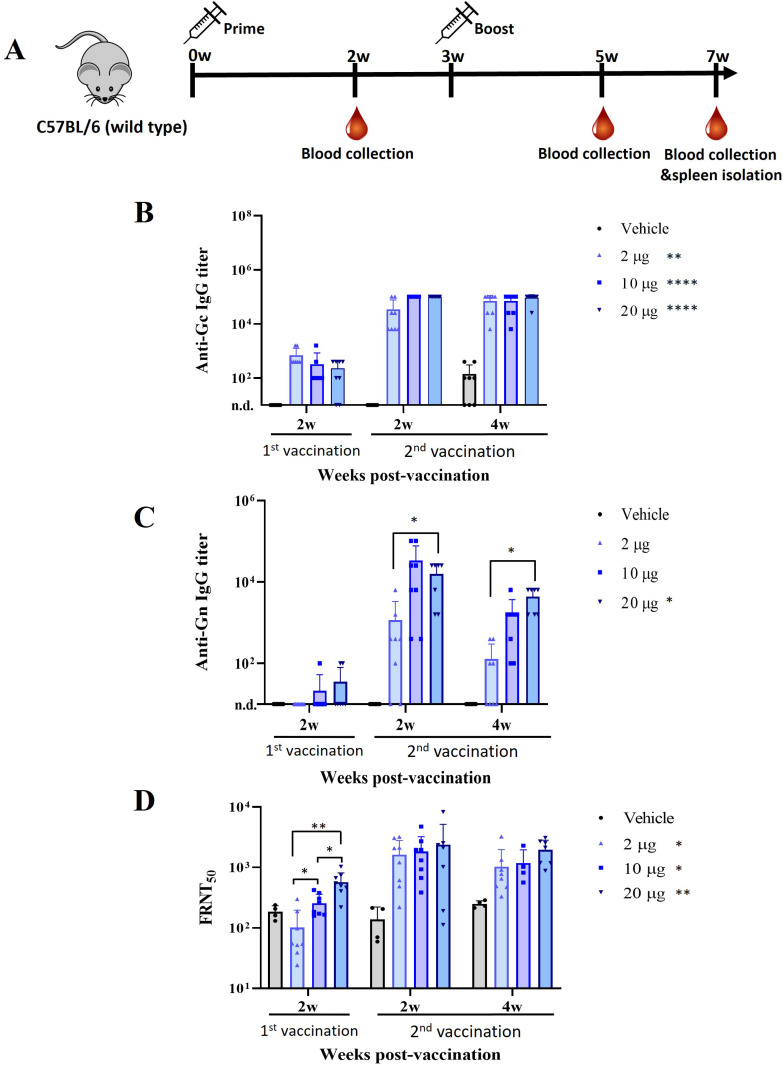
Immunogenicity of SFTSV mRNA-Gn-Gc vaccine candidates in mice. **(A)** Mouse immunization and blood collection schedule. Wild type C57BL/6 mice were immunized with three doses (2, 10, and 20 μg) of mRNA-Gn-Gc vaccine (VER-001). **(B)** Anti-SFTSV Gc specific IgG response was measured by ELISA. **(C)** Anti-SFTSV Gn specific IgG response was measured by ELISA. The serum samples were collected from immunized mice (*n* = 8) at two weeks after the first and second immunization. Vehicle is represented Empty LNP. Cut off (mean O.D. + 5.076 × S.D) was determined using unvaccinated mice. **(D)** Neutralizing activity of immunized mice serum (*n* = 8) to SFTSV determined based on FRNT_50_. Mice sera were collected at two weeks and four weeks after 1^st^ and 2^nd^ immunization. Error bar, mean ± S.D.; P value calculated by the t-test and 2way ANOVA. *p < 0.05, **p < 0.01, ***p < 0.001, and ****p < 0.0001. This figure was created using Openclipart.

To evaluate immunogenicity in VER-001 immunized mice, female 8-week-old C57BL/6 mice were intramuscularly immunized with three doses of the vaccine candidates at three-week intervals. Serum samples from immunized mice were collected 2 weeks after each immunization and sera were collected 1 month after the last booster. The levels of SFTSV antigen (Gn and Gc)- specific total IgG in the sera of mice immunized with VER-001 were detected using ELISA. All immunized mice showed a significant increase in antibody response to the Gc antigen after each of the three immunization doses (Fig 2A). Specifically, Gc-specific total IgG responses elevated to a high level after the first immunization, increased further at 2 weeks of immunization, and remained elevated up to 4 weeks after the second immunization. However, the antibody response to the Gn antigen in mice immunized with the 20 µg dose showed a significant increase (Fig 2B). Unlike Gc-specific total IgG responses, Gn-specific total IgG responses did not increase after the first immunization at any of the three doses but exhibited a significant rise after the second immunization in the 10 µg and 20 µg groups (Fig 2C). Mice immunized with the vehicle (empty LNP) showed no detectable levels of SFTSV antigen-specific IgG.

We examined the neutralizing antibody generation capacity against the SFTSV B genotype, 2015-JJ01 strain, in the sera of mice immunized with VER-001 to assess its effectiveness. A virus neutralization assay was performed using the sera from mice immunized with VER-001. VER-001 exhibited robust virus neutralization activity in all immunized mouse groups in a dose-dependent manner, whereas no virus neutralization activity was detected in mice immunized with vehicle only after the first immunization (Fig 2D). Furthermore, the FRNT_50_ titers of 2 μg, 10 μg, and 20 μg significantly enhanced and were sustained after the second immunization, respectively. Thus, VER-001 induced a stronger immune response, with higher Gn- and Gc-specific IgG titers than the vehicle and a high neutralizing antibody generation response in wild-type C57BL/6 mice.

### VER-001 induced antigen specific T cell response in immunized mice

**Fig 3:**
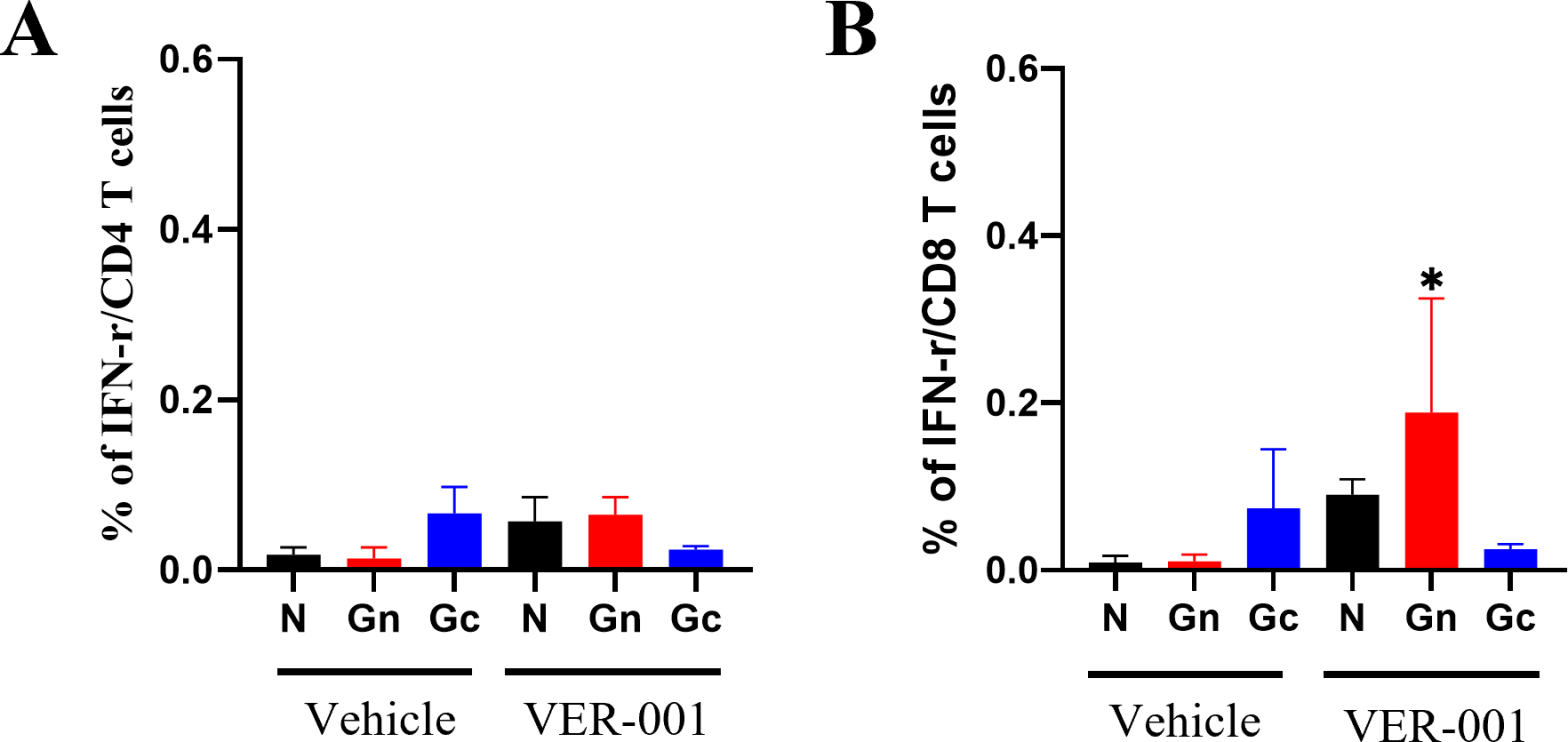
Generation of SFTSV antigen-specific T cell response in immunized WT mouse with mRNA-Gn-Gc. T cell response was analyzed using flow cytometry after SFTSV-specific antigen, Gn and Gc stimulation. Gating results were summarized to IFN-γ producing CD4 (A) and CD8 (B) positive T cell response from mRNA-Gn-Gc vaccinated WT mouse (*n *= 5). Error bar, mean ± S.D.; P value calculated by t-test. *p < 0.05.

To further investigate the specific immune response against SFTSV, we performed flow cytometry to assess the T cell response. The mice were immunized at three-week intervals. To analyze the T-cell response, splenocytes from mice immunized with high dose of VER-001 were collected 2 weeks after the final immunization. The frequency of IFN-γ-producing CD8^+^ T cells in response to Gn antigen showed a significant increase in the groups of mice immunized with VER-001 compared to the mock-immunized mice (Fig 3B). However, the frequencies of IFN-γ-producing CD4^+^ or CD8^+^ T cells in response to Gc were not significantly different between the immunized mice and the control group (Fig 3). Our findings demonstrated the significant role of Gn in inducing effective T cell responses against SFTSV antigens. Moreover, VER-001 induced SFTSV-specific cellular immunity against antigens.

### VER-001 confers complete protective efficacy in IFNAR^-/-^ mice

**Fig 4:**
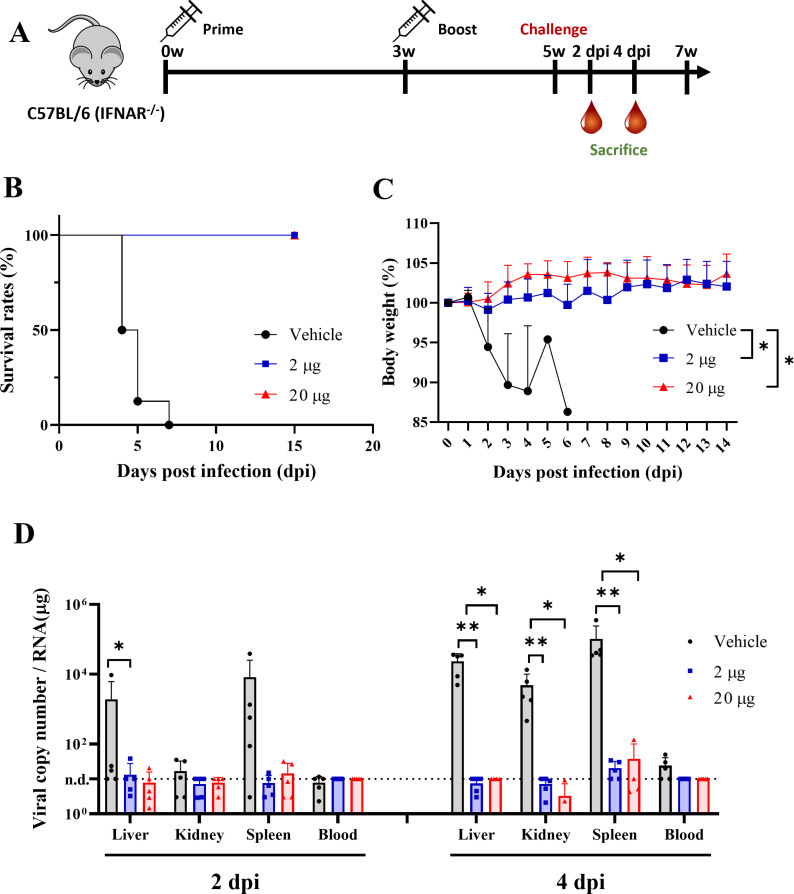
Complete protective efficacy of VER-001 in IFNAR^-/-^mice. **(A)** Mouse immunization and challenge schedule. IFNAR^-/-^ based C57BL/6 mice were immunized with two doses (2 and 20 μg) of the mRNA-Gn-Gc vaccine (VER-001), administered twice at 3-week intervals, and then challenged with 1 × 10^3^ PFU of SFTSV. **(B)** Survival rate, (C) body weight changes of immunized mice with VER-001 (low dose: *n* = 8, blue line, high dose: *n* = 8, red line) and empty LNP (control, *n* = 8, black line) group after lethal SFTSV challenge. IFNR^-/-^ mice were challenged subcutaneously with 10^3^ PFU of SFTSV at two weeks after last immunization. **(D)** Viral copy numbers from organs (liver, kidney, and spleen) and whole blood of mice immunized with VER-001 (low dose: *n* = 8, blue line, high dose: *n* = 8, red line) and empty LNP (control, *n* = 8, black line) group at 2 dpi and 4 dpi. Error bar, mean ± S.D.; P value calculated by the two-tailed Mann-Whitney U test and 2way ANOVA. *p < 0.05, ** *p *< 0.01. This figure was created using Openclipart.

To evaluate the protective efficacy of VER-001, IFNAR^-/-^ mice were immunized with VER-001 at 3-week intervals. Two weeks after the last immunization, mice were subcutaneously challenged with lethal SFTSV-2015-JJ01 (1 × 10^3^ PFU) (Fig 4A). The survival rate and body weight of the vaccinated mice were monitored for 2 weeks after virus challenge and clinical signs were confirmed. All VER-001-immunized mice survived and no decrease in body weight was observed (Fig 4B and 4C). In contrast, all vehicle-immunized mice died between 4 and 7 days after SFTSV challenge and rapidly lost body weight (Fig 4B and 4C). Viral loads significantly reduced in all mice immunized with VER-001 compared to those administered the vehicle only at 2 and 4 dpi. Specifically, viral RNA was undetectable in the blood (at 2 and 4 dpi) and liver (only at 2 dpi) of mice immunized with VER-001 (Fig 4D). To assess the differences in efficacy between low and high vaccine doses, two groups of mice were studied. However, no significant differences were observed between the groups in terms of body weight, survival rate, or viral load (Fig 4D). This indicates that a low dose of VER-001 provided complete protective efficacy comparable to that of a high dose of VER-001. We evaluated the pathogenicity of challenged mice following immunization with VER-001 or vehicle (Table 1). All groups showed minimal infiltration of inflammatory cells and hepatocyte necrosis in the liver (S2 Fig). The white pulp of the spleen of mice immunized with vehicle and a low dose of VER-001 showed mild-to-moderate lymphocyte necrosis, inflammatory foci, and an increase in mononuclear cells (S2A Fig). We further assessed the kidney pathogenicity based on blood biochemical parameters. However, no significant differences in immunization with VER-001 or vehicle were observed. (S2B Fig). Thus, VER-001 reduced viral replication and completely protected IFNAR^-/-^ mice against lethal SFTSV.

**Table 1:** Pathology scores of mice immunized with VER-001 and PBS.

Organs	Findings	Distribution	2 dpi			4 dpi		
			Vehicle (*n* = 3)	Low (*n* = 3)	High (*n* = 3)	Vehicle (*n* = 3)	Low (*n* = 3)	High (*n* = 3)
**Liver**	Infiltration of inflammatory cell	Focal	1 (±^*^)	1 (±)	0	0	1 (±)	0
		Multifocal	0	0	1(+)	1 (+)	2 (±, ±)	2 (±, ±)
	Necrosis	Focal	1	0	0	0	0	0
		Multifocal	0	0	0	1	0	0
**Spleen**	Decreased cellularity in the white pulp	Diffuse	0	1 (±)	0	1 (+)	0	0
**Kidney**	–	–	–	–	–	–	–	–

* Severity grade: ± , minimal; + , slight.

## Discussion

An equal mixture of two mRNAs expressing the full-length SFTSV glycoproteins, Gn and Gc, was used. This approach reconstitutes an intact SFTSV viral envelope structure, enabling the induction of immune responses similar to those triggered by natural viral infections. VER-001 successfully generated Gn/Gc-specific antibodies with high SFTSV neutralization ability even after the first immunization. Additionally, neutralizing antibody levels were dose-dependent after the first immunization. However, after the second immunization, neutralizing antibody levels consistently maintained their peaks without dose-dependent differences. Thus, an mRNA vaccine dose as low as 2 µg could provide sufficient efficacy and could also help reduce costs in the future production process.

An mRNA SFTSV vaccine candidate expressing only Gn of SFTSV was previously developed [56]. An mRNA vaccine encoding Gn of SFTSV elicited high immunogenicity and completely protected from lethal dose of SFTSV. Some vaccinated mice displayed mild weight loss, supporting the possibility that the SFTSV Gn antigen could be a suitable antigen for SFTSV vaccine development. Another SFTS Gn-head mRNA vaccine successfully generated antibodies and fully protected against SFTSV [20]. Both the mRNA vaccines were composed only of the Gn of the SFTSV glycoprotein, in contrast to our full-sequence Gn/Gc vaccine. Moreover, all immunized mice experienced weight loss after challenge infection, but in our experiment, no weight loss was observed even at low dose (2 μg). These findings suggest that VER-001 contributes to the reduction of clinical symptoms after viral challenge in a mouse model.

A limitation of our study is the lack of confirmation of the long-term durability of VER-001. An mRNA vaccine encoding the SFTSV Gn antigen demonstrated the durability of the mRNA vaccine through high IgG antibody titers and neutralizing antibody levels maintained until 12 weeks after the 2^nd^ immunization [23]. Therefore, confirming VER-001 durability is crucial for future mRNA vaccine studies. Second, our study used IFNAR^-/-^ mice, which lack type I interferon receptor function, to evaluate their protective ability rather than for immune response assays. However, we have already observed comparable humoral and cellular immune responses between IFNAR^-/-^ and wild-type C57BL/6 mice, indicating that IFNAR^-/-^ mice elicit similar immune response dynamics following vaccination with the SFTSV DNA vaccine or subunit vaccine [16,19]. Third, the present study utilized structural antigens derived from SFTSV genotype F (KAGWH3). The challenge virus was 2015-JJ01, which belongs to genotype B. The genetic diversity of SFTSV may affect disease severity, and the efficacy of protection could vary depending on the vaccine antigens and challenge viruses derived from different strains [57,58]. Although a challenge study was not performed for each SFTSV genotype, a neutralizing antibody assay was conducted using SFTSV genotypes B, D, and F. Sera obtained from mice immunized with VER-001 exhibited equivalent levels of neutralizing activity against viruses of genotypes B, D, and F (S3 Fig). This result suggests that VER-001 exhibits cross-neutralizing activity in a mouse model and may have potential cross-protective abilities against diverse SFTSV strains. However, as currently there are no results on cross-protective efficacy, accessing cross-protection across various genotypes is necessary.

In summary, this study demonstrated that our SFTSV mRNA vaccine candidate can effectively protect against lethal SFTSV infection by rapidly inducing antibody formation and SFTSV antigen-specific T cell responses in mice. These data support the use of VER-001 as an SFTSV vaccine in future human trials.

## Supporting information

S1 Fig Gating strategies for T cell analysis using flow cytometry. CD4 and CD8 positive T cell responses on splenocyte from high dose (20 μg) of VER-001 immunized mice using FACs.
(TIF)

S2 Fig Histopathological findings in organs of mice infected with SFTSV. (A) and (B) histopathology of the liver and spleen from SFTSV infected mice through H&E staining at 2 and 4 dpi, comparing vehicle, low dose (2 μg), and high dose (20 μg). The yellow arrow indicates hepatocellular vacuolar degeneration and necrosis. 40 × and 200 × magnifications.
(TIF)

S3 Fig Confirmation of cross-reactivity through virus neutralization assays of various genotypes of SFTSV. C57BL/6 mice were immunized with 20 μg of VER-001 twice. Cross-reactivity was confirmed using the FRNT_50_ using serum from immunized mice one-month post-vaccination with the three genotypes (B, D, and F) of SFTSV.
(TIF)
