# Supplementary figures and images for: The immunogenicity and protection efficacy evaluation of mRNA vaccine candidate for severe fever with thrombocytopenia syndrome in mice
Source: PLoS Negl Trop Dis. 2025 Apr 30;19(4):e0012999. doi: 10.1371/journal.pntd.0012999 (PMC12068709; doi:10.1371/journal.pntd.0012999)

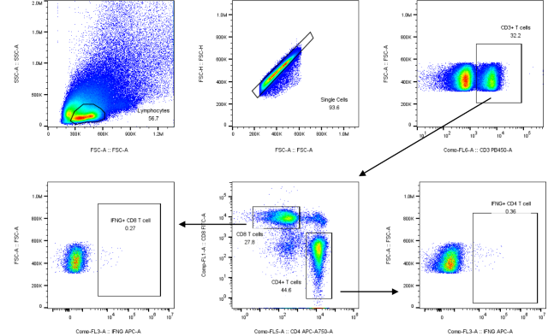

Supplement: S1 Fig — CD4 and CD8 positive T cell responses on splenocyte from high dose (20 μg) of VER-001 immunized mice using FACs. (TIF) [file pntd.0012999.s001.tif]

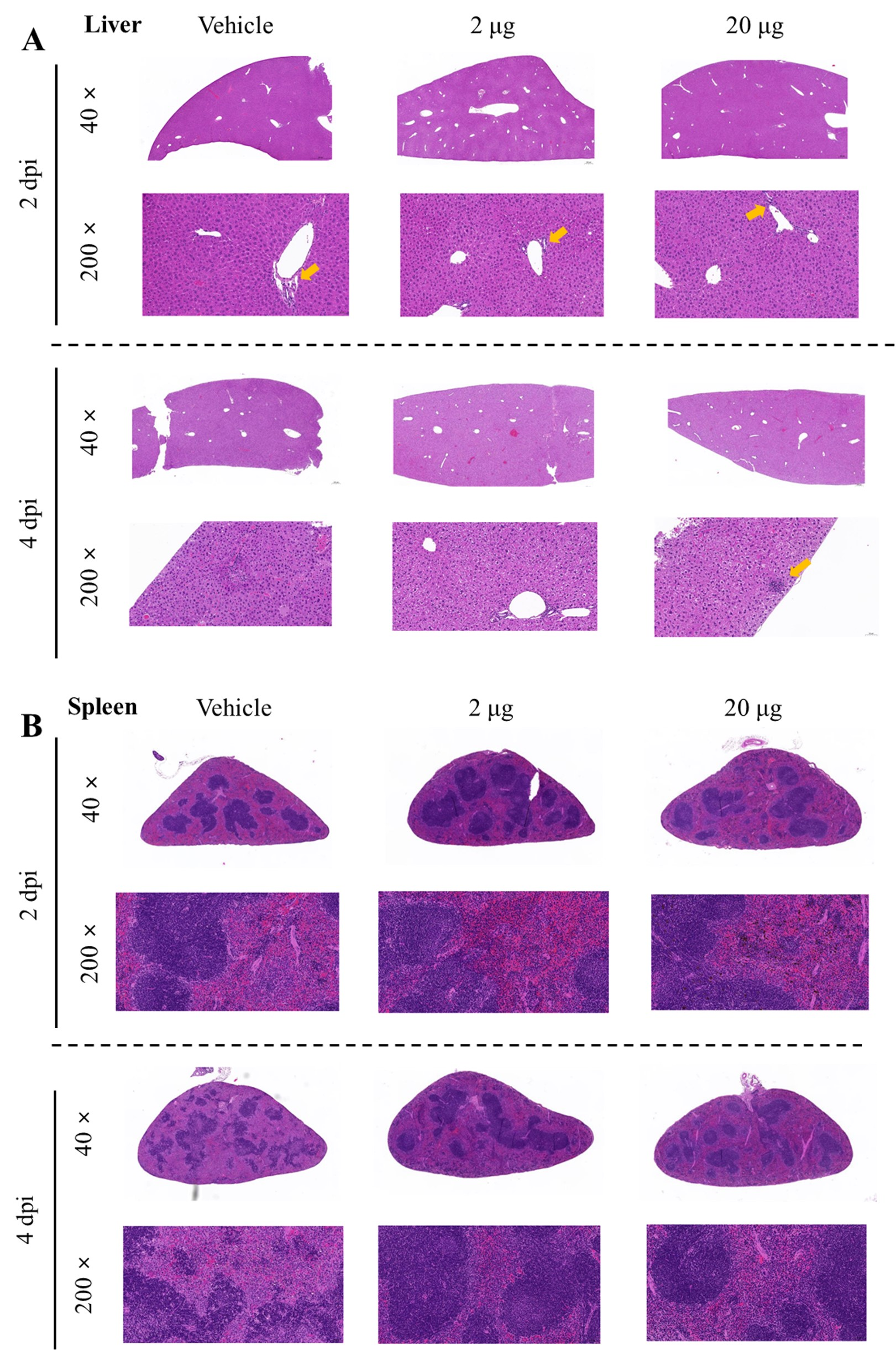

Supplement: S2 Fig — (A) and (B) histopathology of the liver and spleen from SFTSV infected mice through H&E staining at 2 and 4 dpi, comparing vehicle, low dose (2 μg), and high dose (20 μg). The yellow arrow indicates hepatocellular vacuolar degeneration and necrosis. 40 × and 200 × magnifications. (TIF) [file pntd.0012999.s002.tif]

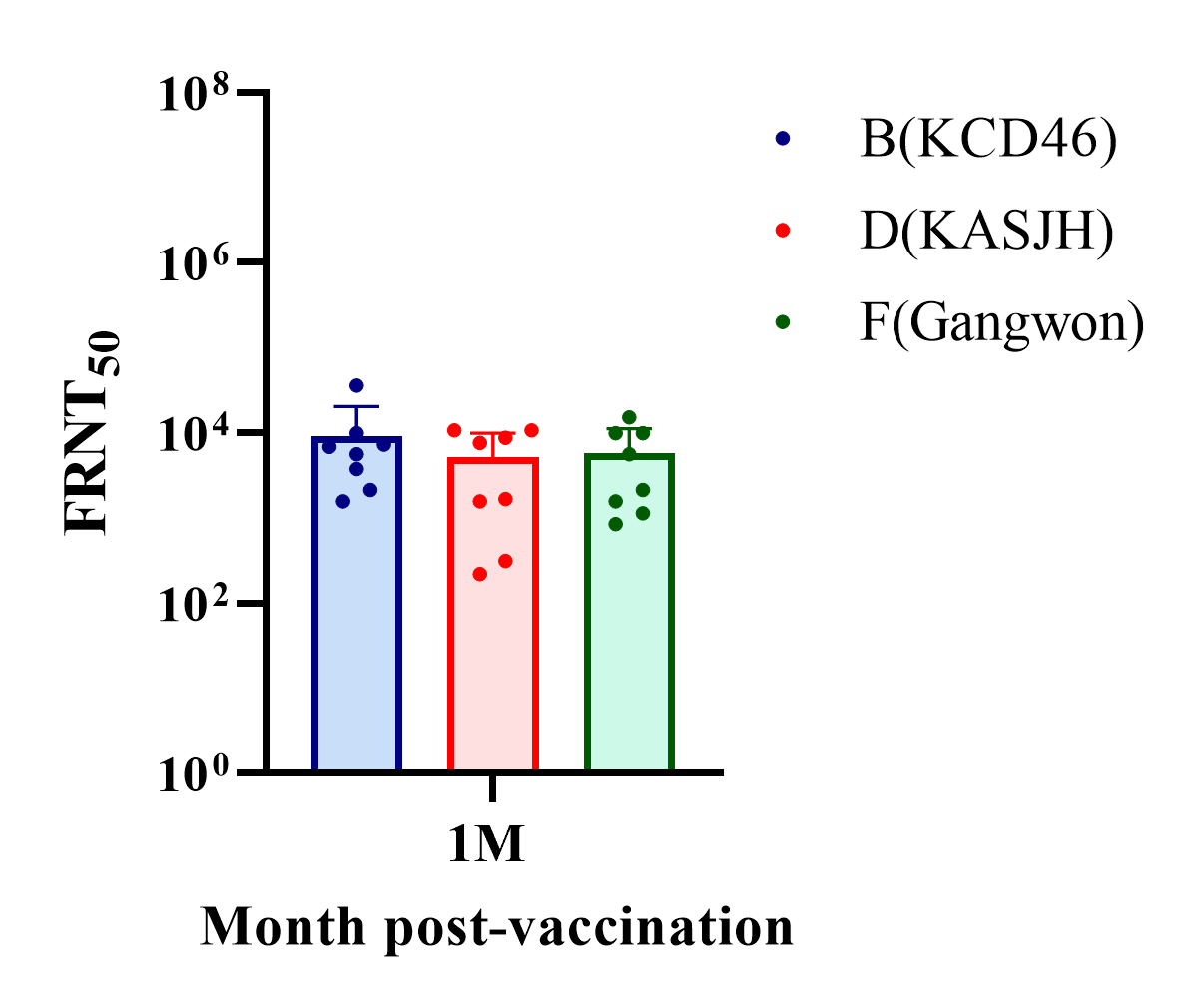

Supplement: S3 Fig — C57BL/6 mice were immunized with 20 μg of VER-001 twice. Cross-reactivity was confirmed using the FRNT50 using serum from immunized mice one-month post-vaccination with the three genotypes (B, D, and F) of SFTSV. (TIF) [file pntd.0012999.s003.tif]
